# Bridging the Local Persistence and Long-Range Dispersal of Highly Pathogenic Avian Influenza Virus (HPAIv): A Case Study of HPAIv-Infected Sedentary and Migratory Wildfowls Inhabiting Infected Premises

**DOI:** 10.3390/v14010116

**Published:** 2022-01-10

**Authors:** Dae-sung Yoo, Sung-Il Kang, Yu-Na Lee, Eun-Kyoung Lee, Woo-yuel Kim, Youn-Jeong Lee

**Affiliations:** 1 Animal and Plant Quarantine Agency, Gimcheon 39660, Korea; shanuar@korea.ac.kr; 2 Avian Disease Division, Animal and Plant Quarantine Agency, Gimcheon 39660, Korea; ksilion@korea.kr; 3 Avian Influenza Research and Diagnostic Division, Animal and Plant Quarantine Agency, Gimcheon 39660, Korea; ynlee27@korea.kr (Y.-N.L.); ensenble@korea.kr (E.-K.L.); 4 Honam National Institute of Biological Resources, Mokpo 58762, Korea; kentish@hnibr.re.kr

**Keywords:** flyways, highly pathogenic influenza virus, home range, migratory bird, tracking device

## Abstract

The past two decades have seen the emergence of highly pathogenic avian influenza (HPAI) infections that are characterized as extremely contagious, with a high fatality rate in chickens, and humans; this has sparked considerable concerns for global health. Generally, the new variant of the HPAI virus crossed into various countries through wild bird migration, and persisted in the local environment through the interactions between wild and farmed birds. Nevertheless, no studies have found informative cases associated with connecting local persistence and long-range dispersal. During the 2016–2017 HPAI H5N6 epidemic in South Korea, we observed several waterfowls with avian influenza infection under telemetric monitoring. Based on the telemetry records and surveillance data, we conducted a case study to test hypotheses related to the transmission pathway between wild birds and poultry. One sedentary wildfowl naturally infected with HPAI H5N6, which overlapped with the home range of one migratory bird with H5-specific antibody-positive, showed itself to be phylogenetically close to the isolates from a chicken farm located within its habitat. Our study is the first observational study that provides scientific evidence supporting the hypothesis that the HPAI spillover into poultry farms is caused by local persistence in sedentary birds, in addition to its long-range dispersal by sympatric migratory birds.

## 1. Introduction

Over the last decade, the increasing number of poultry holdings around the globe has been severely affected by the highly pathogenic avian influenza (HPAI) virus. The periodic emergence of the novel HPAI virus weakens efforts of prevention and control for epidemics. Indeed, in Asia, two clades, clade 2.3.4.4A (2014–2015) and clade 2.3.4.4B (2016–2020), were dispersed by wild birds, causing outbreaks in poultry on an unprecedented scale [1]. Moreover, since 2003, South Korea has experienced consecutive HPAI epidemics caused by three different virus subtypes (H5N1, H5N8, and H5N6).

In the expected emergence of a novel HPAI virus epidemic, it is crucial to identify potential transmission mechanisms of HPAI in poultry holdings to develop an intervention strategy. Wild waterfowls are a natural reservoir of the avian influenza virus (AIv), and play a primary role in the dispersion of the virus across the nation’s poultry holdings. A previous study highlighted that sedentary waterfowl species play an essential role in the local persistence of HPAIv and transmission to domestic poultry [2]. A further study reported a spatiotemporal relationship between local persistence and long-range dissemination of AIv among wild bird populations [3], hypothesizing that the pathogen transfer from migrant to sedentary wild birds occurs through interspecies contact at wintering sites. Another study estimated that the mean of the infectious period for the HPAI virus in a long-distance flying migrant is only 5–15 days per year [4], indicating that migratory birds have a biological limitation in directly delivering the virus to domestic poultry.

In South Korea, migratory birds generally migrate for winter from September to December, returning to breeding sites from mid-February to May. During the active surveillance for AIv in South Korea during those periods, the dabbling duck (Anatinae), especially mallard and spot-billed duck, is repeatedly reported as having HPAI virus infection, irrespective of subtypes [5]. Additionally, the HPAI virus was further identified in those species in February and March, which is not considered the migration season. This observation suggests that there is a persistence of the HPAI virus among local wild bird species, which might originate from the migratory birds. Moreover, according to epidemiological reports on the HPAI epidemic in South Korea, wild birds were identified as the likely source of HPAI infections in several infected premises because of the close distance to localities where the virus was identified in the wild bird samples [6]. Therefore, in order to plan for efficient prevention and timely preparedness, it is necessary to scrutinize the relationship between the local circulation of HPAI in the wild bird populations and its occurrence in poultry holdings. However, none of the observational studies support the hypothesis regarding the interplay of the HPAIv transmission dynamics between sedentary wildfowl and migratory birds associated with occurrences in poultry holdings.

During the 2016–2017 HPAI epidemic, one spot-billed duck was identified as testing positive for the HPAI H5N6 antigen, while four other spot-billed ducks were positive for the H5-specific antibody. The movement trajectory data of those waterfowls were monitored via global positioning system (GPS) devices and were attached as part of the monitoring projects for HPAI prevention purposes [7]. Moreover, a few infected poultry holdings that were temporally and geographically close were placed in the localities where the HPAI antigen and antibody were identified in those waterfowls. This was the first observational case that would contribute to enlightening the elusive pathways related to HPAI viral introduction, transmission, and proliferation among wild and farmed birds.

In this regard, we first aimed to demonstrate the interplay between migratory wildfowl and sedentary wildfowl by investigating the home range of waterfowl. Here, we utilized the probability overlap of utilization distribution (UD), or the probability distribution, by defining the animal’s use of space and movement patterns using the recorded geocoordinates of the five AIv-infected waterfowls. Additionally, a phylogenetic analysis was conducted to assess the genetic association between HPAIv-infected sedentary waterfowl and the poultry located inside its home range, testing the hypothesis on the potential HPAI transmission pathway from wild to domesticated birds. This case study provides a genuine perspective on the potential transmission pathway associated with the HPAI virus being introduced to local poultry productions by wild birds.

## 2. Materials and Methods

### 2.1. Avian Influenza Outbreak Data

From 2016 to 2017, an active surveillance program for HPAI was initiated both for domestic poultry holdings and wild birds. This program targeted 32,195 poultry holdings for antigen tests and 9959 farms for antibody tests [6,8]. Similarly, 77,960 samples were collected from wild birds (carcass or cloacal and oropharyngeal swabs for captive feces) at approximately 3000 sites for an HPAI antigen test, while 4416 samples were taken from wild birds in about 300 places across the country for an HPAI antibody test [6,8].

**Figure 1:**
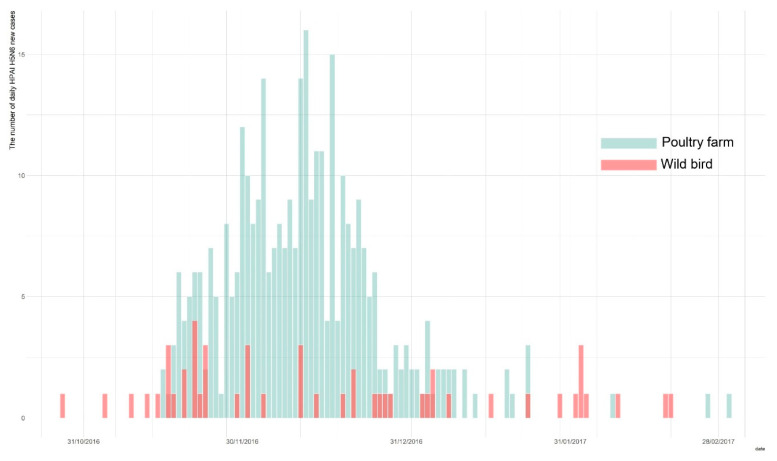
The epidemic curve of highly pathogenic avian influenza (HPAI) outbreak in wild bird populations (blue) and poultry holdings (red) during the 2016–2017 epidemic.

Owing to the active surveillance of domestic poultry holdings and wild birds during the 2016–2017 HPAI H5N6 epidemic in South Korea, a total of 394 total cases were identified, with 343 cases in poultry holdings, and 51 confirmed in the wild bird populations [6,9]. The first wild bird HPAI H5N6 case was reported on the 28 October 2016, and was subsequently followed by the first HPAI occurrence in poultry farms on the 16 November 2016. The last report of a new infection was on the 3 March 2017. We obtained both the wild bird species and poultry farms data on HPAI cases from the Korea Animal Health Integrated System (KAHIS, accessed on 1 December 2019) (Figure 1).

### 2.2. Telemetry Records for Wild Waterfowls

Since 2013, wild bird species such as the spot-billed duck and mallard have been fitted with GPS tracking devices, using GPS-mobile phone-based telemetry (WT-300), manufactured by the Korea Institute of Environment Ecology. For each bird, the GPS device is attached following capture and after a sample has been taken via an oropharyngeal and cloacal swab for the HPAI test. Each GPS transmitter was programmed to transmit its geographical coordinates every 4 h within a 10 to 40 m range error [10,11]. Following these protocols, during the epidemic, the HPAI H5N6 antigen and H5-specific antibody were identified in one spot-billed duck and in four other spot-billed ducks, respectively. Those birds had a series of observational records through GPS tracking devices (Table 1). The HPAI H5N6-infected spot-billed duck (*Anas poecilorhyncha*), identified as “sb1601”, had its oral and cloaca samples taken, and a GPS tracking device attached on 12 December 2016. Following this, it continued to transmit its geo-location data until the 10 August 2017. Additionally, four H5-specific antibody-positive spot-billed ducks (“sb1602”, “sb1603”,” sb1604”, and “sb1605”) were similarly fitted with individual GPS transmitters on 12 December 2016, after undergoing the aforementioned sampling procedures. Their first location signal was received on 17 December 2016, while the dates of their last signal were 26 March 2017, 24 April 2017, 15 July 2017, and 23 September 2017, respectively. We used this post-infection movement to analyze the sympatric wildfowl population’s transmission dynamics. The GPS data for those waterfowls were obtained from KAHIS (accessed on 11 March 2020).

## 3. Methods

### 3.1. Study Framework

**Figure 2:**
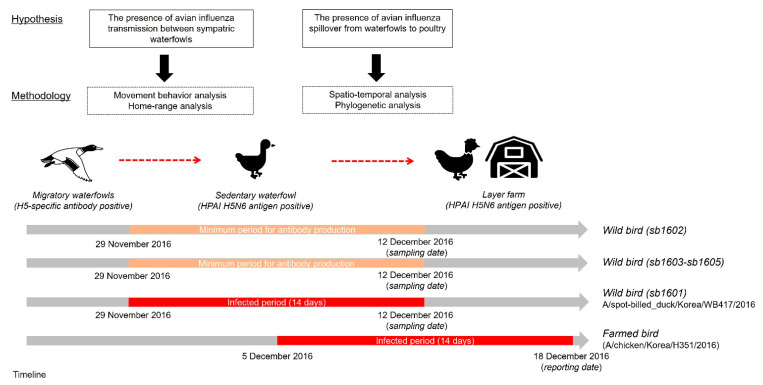
Study framework for testing the hypothetical relationship of wild birds, including migratory and sedentary waterfowl, alongside poultry farms. The red square represents the maximum infected period, assumed to have been 14 days before the sampling date, while the bright ivory denotes the duration of antibody detection, assumed to start from at least 14 days before the detection date.

Figure 2 presents an overview of the study framework related to our hypotheses on the spillover of AIv and the corresponding techniques used to test those hypotheses. First, we investigated HPAI-infected and H5-specific antibody-positive spot-billed ducks’ movement trajectories to categorize movement behavior. Then, the home range of those five spot-billed ducks was estimated to observe the movement characteristics and evaluate spatial interactions amongst them. Next, we examined the spatiotemporal relationship between one HPAIv H5N6 infected waterfowl and domestic layer farms located within the corresponding home range using cluster and phylogenetic analyses. This allowed us to test the hypothesis that the HPAI virus initially disseminated from wild birds before potentially spreading to farmed birds.

### 3.2. Movement Characteristics of Avian Influenza-Infected Wild Birds

We tested the hypothesis that avian influenza transmission pathways amongst wildfowl species consisted of long-range dispersal by migratory birds and intra-species spread to indigenous sympatric waterfowl. Therefore, to classify the movement modes into five criteria [12], including migratory, mixed migratory, disperser, home range, and nomadic (or sedentary), we first calculated a behavior net squared displacement (NSD) of the five wild birds with an AIv infection. NSD is the analytical technique used for the identification of different behavioral modes in wild animals. These are typically obtained from a series of locations by measuring the square of the Euclidean distance between a given location and the putative origin of a movement path [13].

Next, we estimated the home range of those birds using dynamic Brownian Bridge Movement Models (dBBMM) to describe the UD as the extent of the distribution of locations of a wild bird. Additionally, the minimum convex polygon method (MCP) was calculated at 99% for home range limits. The MCP here refers to the area that the animal inhabited according to a minimum area polygon, which contains all their observed locations. Moreover, because the movement data generated from GPS telemetry are highly likely to be autocorrelated in space and time by nature, trajectory-based estimation approaches such as dBBMM represent a more suitable method, as they account for any temporal autocorrelation and spatial uncertainty in the sampled data. The dBBMM was introduced as an alternative to kernel density methods, which use individual sampled locations to calculate home range without the temporal structure of data. This method is either likely to result in over-smoothing for small data sets or under-smoothing for large data sets because it uses an animal’s movement path with a compromised time interval and distance between consecutive locations to estimate UDs, based on heterogeneous Brownian motion variance [14]. The 50% utilization of distribution corresponding to the core home range was calculated with a 99% confidence interval by dBBMM.

Furthermore, we assessed the spatial interaction between migratory and sedentary birds with respect to the possibility of intra-species viral transmission. Thus, the overlap index representing the probability overlap of UD and distance between two individuals [15], in addition to the volume of intersection under the full UDs of two paired spot-billed ducks, was estimated to calculate the probability of being in contact with either of the paired wild birds. Net squared displacement and overlap index estimation was conducted using the adehabitatHR package (version 0.4.16.) in R software 4.1.1 [16]. The home range was estimated using the adehabitatLT package (version 0.3.24.) [17]. Spatial visualization of the analytic outputs was performed with ArcGIS 10.8.1. (ESRI Co., Redlands, CA, USA).

### 3.3. Interaction Analyses between Poultry and Wild Birds

According to a previous study [18] on the 2016–2017 HPAI epidemic, spatiotemporal clusters were detected during the 2016–2017 HPAI epidemic using phylogenetic cluster information of HPAI H5N6 virus-infected premises, where five different genotypes (C-1 to C-5) of clade 2.3.4.4C HPAIv were identified from the homologies of the PA and NS genes [19,20]. The spatiotemporal cluster identified in that study indicated that a particular genetic type of HPAIv (i.e., C-2) occurred significantly at a higher rate inside the cluster during a specific period than any other genetic sub-group HPAIv. There were two spatiotemporal clusters detected for HPAI H5N6 infection in poultry farms. The first (Cluster A) was a mid-region in South Korea with a 54.25 km radius from 19 November 2016 to 13 January 2017, where the C-2 phylogenetic group of HPAIv outbreak in poultry farms predominantly occurred with 5.03 relative risk (*p* < 0.01) compared to any other phylogenetic group of viruses. The second (Cluster B) was a southern region primarily with a C-3 phylogenetic group of HPAIv infection in poultry farms, with a relative risk of 35.40 within a 19.62 km radius from 26 November 2016 to 6 January 2017. Using the outcomes from the spatiotemporal cluster analysis, we located the home range of one HPAI-infected waterfowl and evaluated the geographical association between its home range and a spatiotemporal cluster of infected premises.

In addition to the geographical interface associated with infected wildfowl and poultry holdings, phylogenetic analysis was performed to examine the genetic similarity between an HPAI-infected waterfowl (virus name ID: A/spot-billed_duck/Korea/WB417/2016, collected 12 December 2016) and an infected premises (virus name: A/chicken/Korea/H351/2016, collected on 18 December 2016) located within its home range. Using RNAs extracted from those isolates that belonged to the same phylogenetic cluster (i.e., C-2), the complete coding nucleotide sequences of hemagglutinin (HA) gene were used to build the tree with MEGA version 6.0 (www.megasoftware.net, accessed on 8 December 2021). To consider the possibility of those HPAI-infected poultry farms being infected from other infected premises belonging to the C-2 genetic sub-group, the isolates from a neighboring infected property (virus name: A/chicken/Korea/H300/2016, collected on 15 December 2016) and two other infected premises (virus name: A/quail/Korea/H641/2016, collected on 29 December 2016, and virus name: A/chicken/Korea/H781/2017, 7 January 2017 [ID: H781], respectively), located in C-2 genotype-prevalent regions, were included in phylogenetic analysis along with the isolates from wild birds (virus name: A/wild_duck/Korea/H793-1/2017, collected on 4 January 2017) that belonged to the same genetic sub-group (Table 1).

## 4. Results

### 4.1. Movement Characteristics

As summarized in Table 2, the utilization of distribution analysis of five avian influenza-infected waterfowls provided a mean moving distance of 943.8 m per 4 h, in addition to a home range of 46.46 km^2^ for a spot-billed duck with a positive HPAIV H5N6 test (i.e., sb1601). The mean distances of three spot-billed ducks (i.e., sb1603, sb1604 and sb1605) with positive H5 antibody tests were 504, 363, and 302 m per 4 h, while they presented a home range of less than 17 km^2^ (16.3 km^2^, 7.55 km^2^, and 3.06 km^2^, respectively). In contrast, one spot-billed duck (i.e., sb1602) flew to North Korea in early May, and thus possessed the highest moving distance of 95.88 km and an estimated 1313.11 km^2^ home range. In addition, behavior net squared displacement (NSD) of those spot-billed ducks indicated one dBBMM, dynamic Brown Bridge Movement model; MCP, minimum convex polygon, sample for HPAI test was taken on 14 December 2016.

One HPAI antigen-positive spot-billed duck (i.e., sb1601) was regarded as sedentary. However, three birds (i.e., sb1603, sb1604, and sb1605) with H5-specific antibodies, a sedentary waterfowl, and one spot-billed duck (i.e., sb1602) with H5 antibodies were indicated as migratory birds.

### 4.2. Geographical Relationship of Avian Influenza-Infected Wild Birds

**Figure 3:**
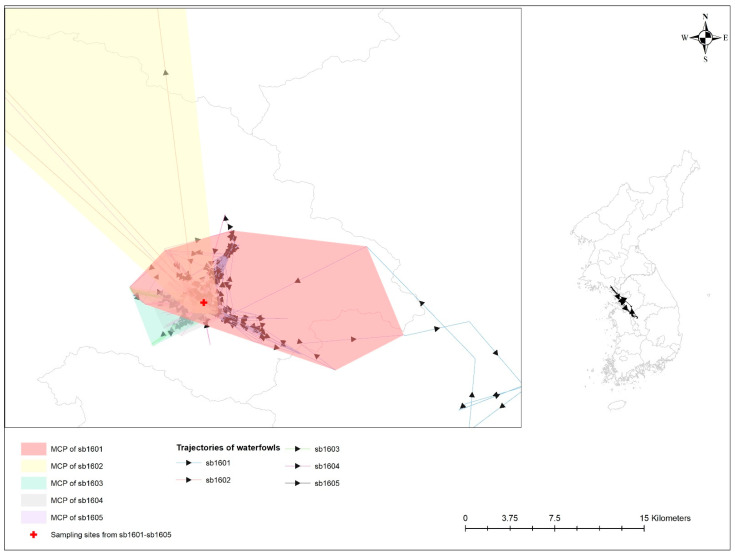
Trajectories and minimum convex polygon (MCP) of five waterfowls with avian influenza infection. Different colors represent 99% MCP of individual waterfowl; bright red for sb1601; bright yellow for sb1602; bright green for sb1603; grey for sb1604; and bright purple for sb1605. Sampling sites for avian influenza antigen and antibody from those birds are denoted by a red cross.

Table 3 provides a statistical probability of the overlap of UD among five spot-billed ducks infected with AIv. All of these birds shared their space with one another and inhabited it at a close distance (see Figure 3). An estimated overlap index, including probability overlap, revealed the probability of the HPAI-infected spot-billed duck (i.e., sb1601) following sedentary bird movement behaviors, such as being in the UDs of other spot-billed ducks with H5-specific antibodies (i.e., sb1602-1604); these values were 0.032, 0.279, 0.173, and 0.430, respectively.

### 4.3. Geographical and Phylogenetic Relationship of Wild Birds to Infected Poultry Premises

**Figure 4:**
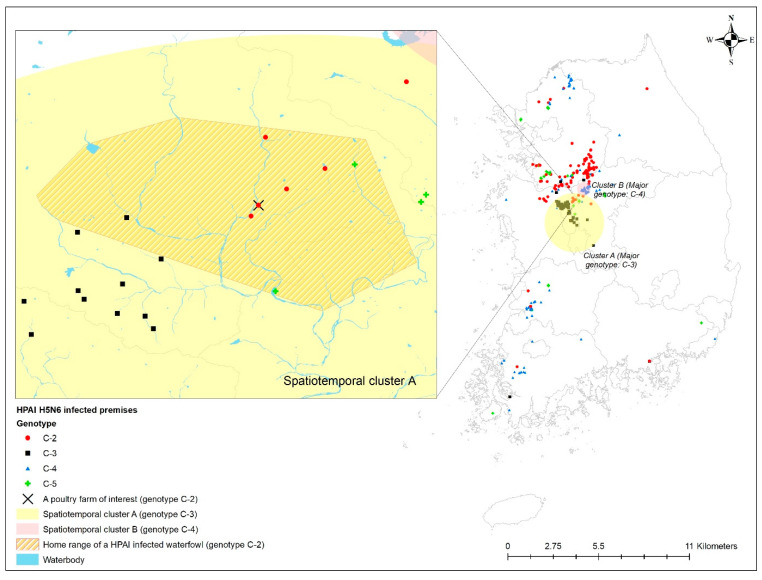
Geographical distribution of the home range of highly pathogenic avian influenza (HPAI) virus-infected premises by genotype, together with spatiotemporal clusters. Enlarged map for the infected chicken farm located in the HPAI-infected waterfowl’s home range.

**Figure 5:**
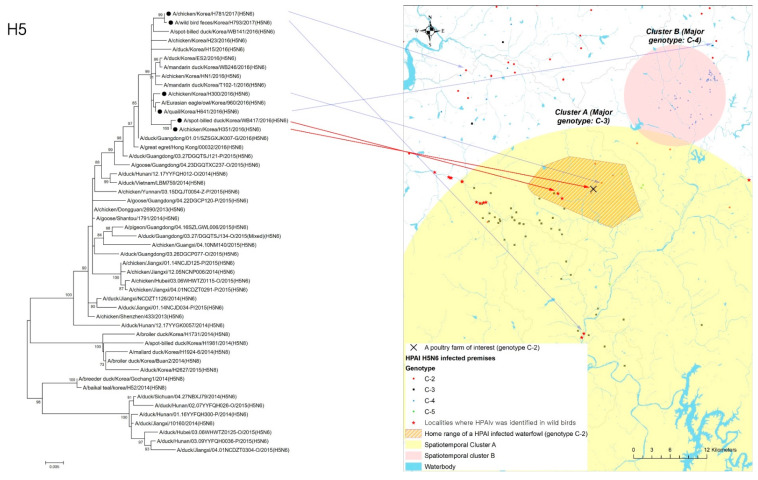
Phylogenetic tree for hemagglutinin (HA) gene of highly pathogenic avian influenza (HPAI) virus isolates from one H5N6-positive spot-billed duck and one chicken farm located inside its home range.Black circle in the tree denotes the isolates from one HPAI-infected waterfowl, one infected premise indicated by a red arrow, a neighboring infected premise, two other infected premises, and another infected wild bird, respectively, located in C-2 a genotype-prevalent area, indicated by a blue arrow.

The home range of an HPAI-infected waterfowl (i.e., sb1601) was in the spatiotemporal cluster, where HPAI H5N6 genotype C-3 infections occurred significantly more than other genotypes in infected poultry farms (Figure 4). Moreover, five poultry farms infected with genotype C-2 were placed inside the spatiotemporal cluster for genotype C-3 and the home range of the infected waterfowl. All isolates examined in this study belonged to clade 2.3.4.4.C. Of them, two infected premises included in the phylogenetic analysis were approximately 500 m away from each other. The phylogenetic analysis found a chicken layer farm (i.e., sample ID = H351) within the MCP of a spot-billed duck (i.e., sb1601, sample ID = WB417), with the HPAI infection showing a closer relationship than isolates from three different poultry farms infected with the C-2 genotype and one wild bird fecal sample (Figure 5).

## 5. Discussion

Over the last decade, an increasing number of HPAI infections have been reported in poultry facilities, sparking huge concern about the emergence of pandemics presenting a substantial threat to both animals and public health [21].The interface of wild and farmed birds in terms of HPAI co-circulation is assumed to be a critical connection impinging on both animal and public health [22]. Thus, we initially conducted an epidemiological case study using data from HPAI surveillance on wild birds and poultry farms. Additionally, telemetry data on wild birds were used to examine the hypothetical association of HPAIv infection in migratory birds and sedentary waterfowls, especially Anatinae, with poultry at the interface of wild birds and domesticated birds [23].

In this study, we found sedentary waterfowls’ potential role as a bridge or liaison host in intermediating HPAIv transmission between sympatric migratory waterfowl species and poultry farms. Furthermore, the results positively correlated with the distribution of HPAI H5N6-infected poultry farms through UD [24], spatiotemporal cluster, and phylogenetic analysis from five spot-billed ducks (*Anas poecilorhyncha*) attached with a GPS tracking device and infected with HPAI H5N6 and positive for H5 antibodies. This suggests that spot-billed ducks could be bridge hosts and/or spillover hosts that share a habitat with maintenance hosts and poultry [24].

More specifically, movement behavior analyses of five spot-billed ducks suggested intra-species transmission of avian influenza. An HPAIv H5N6-infected bird, as well as three other birds with H5-specific antibodies, had a high tendency to be a sedentary or nomadic bird. Alternatively, the remaining one spot-billed duck with H5 antibodies was regarded as a migratory bird that flew to North Korea in early spring, approximately 180 km away from its core habitats, followed by stopping signals for unknown reasons. Importantly, they shared more than 3% of UDs in four resident H5 antibody-positive spot-billed ducks and one HPAIv-detected spot-billed duck during the winter season, leading to possible direct or indirect AIv transmission among them, despite the variations in the spot-billed ducks’ movement patterns.

Indeed, sharing the habitat between migratory and sedentary sympatric wild birds is critical to introducing a novel pathogen into a new area to start local transmission. A previous study suggested a period of only 5–15 days per year when infections from migratory species could result in the dispersal of the HPAI H5N1 virus over a range of 500 km [4]. When migratory waterfowls arrive in the Korean peninsula, it takes an estimated 3–4 days to shed the virus. Wild birds barely become infected with the HPAI virus during migration, assuming the infection takes place in either breeding sites prior to migration or stopover sites, such as Russia and China. Moreover, the recent landscape change in northeastern China into abundant rice paddy areas represents an ideal condition for disease propagation driven by the large recruitment of immunological naïve juvenile birds [25]. Indeed, the C-2 phylogenetic group of viruses (three genomes) shares high NA identities with the Chinese H5N6 viruses that circulated in Guangdong province in early 2016 (98.42–99.78%) [20]. Another phylogenetic analysis indicated that avian influenza is possibly spread from Siberia by migratory birds [26].

According to the World Organization for Animal Health, the onset of the HPAI infection could be at the maximum of 14 days before PCR confirmation. In addition, the antibody response to the pathogen generally starts 14 days post-infection. Considering the timeline to antibody production and infected period, it could be suggested that the spot-billed duck with antibodies could have been infected on 29 November 2016 at the earliest, followed by the onset of infection of the spot-billed duck with the HPAI antigen but no antibodies. Moreover, three sedentary H5-specific, antibody-positive waterfowls sharing UDs in the study allowed us to infer the local circulation or persistence in that area before the H5N6 antigen-positive spot-billed duck had a possible secondary infection. In this circumstance, the viral propagation between those waterfowls could be established by indirect transmission. In other words, considering the environmental persistence of the pathogen observed in this study, particularly in water bodies, it is feasible that the viral diffusion among the sympatric dabbling ducks occurs through sharing the same space. 

Waterfowl species are also known to replicate and shed the HPAI virus for several days without any apparent clinical signs, or before the onset of illness [27]. A recent study reported that the avian influenza virus generally shows significant affinity to the colon of the Anas duck species, indicating a fecal–oral transmission route in these species [28]. According to another previous study, waterfowls with natural low pathogenic avian influenza virus infections performed fewer regional movements than uninfected individuals [29]. Moreover, resident wildfowls are more likely to spread the virus quicker, among those never infected before, than migratory waterfowls immunized against a similar virus [30]. This indicates that some wildfowl could transmit the HPAI virus during a period of asymptomatic infection through indirect as well as direct transmission, similar to the HPAIV H5N6-infected spot-bill duck, which does not show critical change in movement and home range.

Spot-billed ducks play a central role in local persistence and maintenance of viral dissemination. Since 2010, the spot-billed duck has been one of the waterfowl species in which the HPAI virus has been continuously confirmed whenever an HPAIV outbreak occurs at poultry farms in South Korea. A winter migratory bird census in Korea between 2016 and 2017 illustrated an increase in the number of spot-billed ducks in January at the Gog-gyo stream (Pung-SEO). However, only one spot-billed duck was infected with HPAI H5N6 in this core habitat, while approximately 26.1 km west of this location, at Sab-Gyo Lake, the number of spot-billed ducks decreased. According to a wildlife survey in South Korea [31], waterfowl, particularly the spot-billed duck, become the dominant species in waterbodies such as lakes, streams, and rivers, despite a decrease in the total populations of wild birds. A further study demonstrated that the increased habitat suitability for the spot-billed duck in South Korea contributed to the risk of identifying the avian influenza virus in wild bird populations at the initial phase of the epidemic, which is associated with outbreaks in poultry holdings [5]. Therefore, it appears that the abundance of spot-billed ducks resulted in an increase in the susceptibility of the poultry farms, especially domesticated bird farms located in a near waterbody, to avian influenza in South Korea. Moreover, the result of the census in 2014 and 2015 showed the same trend, suggesting these movement patterns would drive an increase in the possibility and frequency of contact between migratory waterfowls and resident spot-billed ducks. This waterfowl species population and direction trend is spatiotemporally correlated with the HPAI virus C-2 genetic sub-group outbreak pattern in poultry farms in 2016. The high-density area of the resident wildfowl may lead to high-risk areas for HPAI infection in poultry farms, as shown in this study. This could be the reason for HPAI-infected commercial layer farms that were located within the home range of one sedentary spot-billed duck with a higher genetic similarity in the phylogenetic analysis.

This case study has several limitations in terms of generalizing findings and reaching a firm conclusion about the transmission pathway from wild to farmed birds. Firstly, the GPS data on the wild birds observed in this study did not cover the whole year or a comparable recording period. Hence, it might bias the outcomes of the movement behavior analysis. However, generally migratory wild birds return to breeding sites from about mid-February to April, when their movements become activated [7,10]. All birds but one continued to transmit the signal until April; notably, the geographical location of the spot-billed duck that was HPAI antigen positive was recorded until August, ensuring the validation of behavior analysis. Secondly, the wild birds observed in this study were captured at a very close distance, which in turn could be regarded as the same herd, hardly distinguishing between migratory and sedentary individuals. However, the time point of capturing those birds was in mid-December, when it was time for the migrant birds to arrive in the wintering area. Additionally, the spot for capturing the birds was a recognized place for wild bird reservoirs where multiple herds of birds were aggregated very densely. Therefore, it could not constrain the possibility of interspecies transmission. Thirdly, no classification criterion for the overlap estimation was commonly available to compare its extent, especially in spot-billed duck species, mainly because of insufficient previous studies. Additionally, since the overlap probability between those birds was estimated using post-infection movements, it could bias inference for potential contact between antibody-positive birds and an HPAI-positive bird because of the vanished shedding period of antibody-positive birds. However, they could have a similar movement and contact pattern before the infection until post-infection movements, given their repeated trajectories and proximity to the capturing site, as mentioned above. Additionally, the phylogenetic tree in this study showed that the virus isolated from HPAI-infected sedentary spot-billed ducks evolved later than the virus isolated from farmed chickens on 18 December 2016. This indicated that it is not feasible to have a spillover from a wild bird into farmed birds. However, we did not incorporate the temporal information to construct a phylogenetic tree owing to the unavailability of collection dates on other sequences used in the study, which could contradict our results. Moreover, even though the phylogenetic tree represents evolutionary change among individual samples, it could also bias the results due to transmission complexities causing a disagreement between the actual transmission history and the phylogeny of the sampled pathogen, such as the presence of within-host variation (i.e., genetically difference of those sampled from the same host) and the possible presence of non-sampled hosts [32]. Therefore, the possibility of spillover from wild birds to poultry still remains. We should interpret the phylogenetic tree with other information related to isolates, such as epidemiological information.

Lastly, inter-farm transmission could be possible because the isolates of all infected premises epidemiologically linked to the poultry of interest in this study (e.g., neighboring farms or connected by vehicle movements) were not included in the phylogenetic analysis due to data availability. Therefore, future work is needed to consider alternative transmission pathway possibilities and other uncertainties. The explicit limitation for generalization of the results from this case study showed that one case of HPAIV infection in wildfowl covered interactions in the transmission of HPAIV between migratory and sedentary waterfowls. This study is the first observational study providing potential evidence of sedentary waterfowl bridging the transmission of highly pathogenic avian influenza between poultry farms and migratory wildfowl.

## Figures and Tables

**Table 1:** Highly pathogenic avian influenza H5N6 isolates used in the phylogenetic study (*n* = 6).

Virus Name	Samples	Collection Date	Latitude/Longitude	GISAID Isolate ID
A/spot-billed_duck/Korea/WB417/2016 ^†^	Oral and Cloacal swab	12 December 2016	36°45′9.550″ N/127°7′0.5052″ E	EPI_ISL_7412777
A/chicken/Korea/H300/2016	Oral and Cloacal swab	15 December 2016	37°47′35.448″ N/127°21′16.6968″ E	EPI_ISL_7412755
A/chicken/Korea/H351/2016 ^‡^	Oral and Cloacal swab	18 December 2016	36°46′30.234″ N/127°19′12.684″ E	EPI_ISL_7412792
A/quail/Korea/H641/2016	Oral and Cloacal swab	29 December 2016	36°59′30.3432″ N/127°38′9292″ E	EPI_ISL_7413688
A/wild_duck/Korea/H793–1/2017	Feces	4 January 2017	36°33′17.1972″ N/127°17′51.468″ E	EPI_ISL_7413520
A/chicken/Korea/H781/2017	Oral and Cloacal swab	7 January 2017	36°55′1.9668″ N/127°4′10.5564″ E	EPI_ISL_7412735

GISAID: Global Initiative on Sharing All Influenza Data. ^†^ denotes the spot-billed duck under the surpervision with telemetry records. ^‡^ denotes the poultry farm located inside the home range of HPAI-infected spot-billed duck.

**Table 2:** Summary of home range and a behavior net squared displacement analysis on spot-billed ducks either with highly pathogenic avian influenza (HPAI) H5N6 virus or with H5-specific antibody.

ID	Test Result	Movement Mode	dBBMM (Unit: km^2^)		MCP (Unit: km^2^)	Date of the First Signal	Date of the Last Signal
			50%	95%	99%		
sb1601	HPAIV H5N6 antigen	Sedentary	2.40	46.46	13.77	17 December 2016	10 August 2017
sb1602	AIV H5 antibody	Migratory	13.46	1313.11	259.75		26 March 2017
sb1603	AIV H5 antibody	Sedentary	0.18	16.30	1.56		24 April 2017
sb1604	AIV H5 antibody	Sedentary	0.01	7.55	1.67		15 July 2017
sb1605	AIV H5 antibody	Sedentary	0.18	3.06	140.94		23 September 2017

**Table 3:** Probability overlaps of the full utilization distributions of spot-billed ducks with highly pathogenic avian influenza H5N6 virus and H5-specific antibody (see Figure 3).

ID	ID (Movement Mode, Type of Positive Test)				
	sb1601 (Sedentary, Antigen)	sb1602 (Migratory, Antibody)	sb1603 (Sedentary, Antibody)	sb1604 (Sedentary, Antibody)	sb1605 (Sedentary, Antibody)
sb1601	1.000	0.032	0.279	0.173	0.430
sb1602		1.000	0.024	0.021	0.006
sb1603			1.000	0.164	0.253
sb1604				1.000	0.151
sb1605					1.000

Values of probability overlap in a range from zero (corresponding to no overlap) to one (corresponding to completely identical utilization distributions).

## Data Availability

The HPAI H5N6 outbreak data can be found here: https://ebook.qia.go.kr/home/view.php?host=main&site=20180221_093341&listPageNow=0&list2Pa-geNow=0&code=0&code2=0&code3=0&optionlisttype=&searchcode=0&searchcode2=0&searchdate=0&searchkey=allsite&searchval=%C1%B6%B7%F9%C0%CE%C7%C3%B7%E7%BF%A3%C0%DA&searchandor=&dummy=&&orders=A (accessed on 20 May 2019).
